# The Characterization of a Biodegradable Mg Alloy after Powder Bed Fusion with Laser Beam/Metal Processing for Custom Shaped Implants

**DOI:** 10.3390/ma17071682

**Published:** 2024-04-06

**Authors:** Doina Raducanu, Vasile Danut Cojocaru, Anna Nocivin, Silviu Iulian Drob, Radu Emil Hendea, Doina Stanciu, Steliana Ivanescu, Vlad Andrei Raducanu, Nicolae Serban, Elisabeta Mirela Cojocaru, Radu Septimiu Campian

**Affiliations:** 1 Department of Metallic Materials Processing and Ecometallurgy, University POLITEHNICA of Bucharest, 060042 Bucharest, Romania; doina.raducanu@upb.ro (D.R.); dan.cojocaru@upb.ro (V.D.C.); nicolae.serban@upb.ro (N.S.); elisabeta.cojocaru@upb.ro (E.M.C.); 2 Faculty of Mechanical, Industrial and Maritime Engineering, OVIDIUS University of Constanta, 900527 Constanța, Romania; 3 Institute of Physical Chemistry “Ilie Murgulescu”, Romanian Academy, Spl. Independentei 202, 060021 Bucharest, Romania; sidrob.icf@gmail.com; 4 Department of Oral Rehabilitation, Faculty of Dental Medicine, Iuliu Hatieganu University of Medicine and Pharmacy, 400349 Cluj-Napoca, Romania; raduhendea@yahoo.com (R.E.H.); rcampian@email.com (R.S.C.); 5 ZIRCON DENT SRL, 400690 Cluj-Napoca, Romania; doinastanciu09@yahoo.com (D.S.); sivanescu@yahoo.com (S.I.); 6 Faculty of Decorative Arts and Design, National University of Arts, 010702 Bucharest, Romania; andrei.raducanu@unarte.org

**Keywords:** Mg alloys, mechanical alloying, laser powder bed fusion for metals, mechanical testing, corrosion resistance

## Abstract

A new Mg-Zn-Zr-Ca alloy in a powder state, intended to be used for custom shaped implants, was obtained via a mechanical alloying method from pure elemental powder. Further, the obtained powder alloy was processed by a PBF-LB/M (powder bed fusion with laser beam/of metal) procedure to obtain additive manufactured samples for small biodegradable implants. A series of microstructural, mechanical and corrosion analyses were performed. The SEM (scanning electron microscopy) analysis of the powder alloy revealed a good dimensional homogeneity, with a uniform colour, no agglutination and almost rounded particles, suitable for the powder bed fusion procedure. Further, the PBF-LB/M samples revealed a robust and unbreakable morphology, with a suitable porosity (that can reproduce that of cortical bone) and without an undesirable balling effect. The tested Young’s modulus of the PBF-LB/M samples, which was 42 GPa, is close to that of cortical bone, 30 GPa. The corrosion tests that were performed in PBS (Phosphate-buffered saline) solution, with three different pH values, show that the corrosion parameters have a satisfactory evolution comparative to the commercial ZK 60 alloy.

## 1. Introduction

Apart from well-known fields of use, such as the automotive field, aerospace and electronics, magnesium alloys are currently widely tested as biomedical components, not only for their high strength-to-weight ratio [1,2], but also for their good structural and mechanical biocompatibility [3,4]. Moreover, in a series of biocompatible materials, magnesium alloys fall under the category of biodegradable materials [5,6], a very useful physical process that prevents a second surgery for removing an anterior inserted bone implant after bone healing due to the fact that magnesium can easily disintegrate in time in human physiological environments [7]. Therefore, if used as temporary orthopaedic implant, novel Mg alloys can replace other bone implants, like Ti-alloys, achieving mechanical performances of human bone by avoiding unwanted stress-shielding effects [8,9,10] and promoting bone remodelling and healing [11,12,13].

The main advantages of using magnesium alloy for bone implants refers to the fact that it exhibits mechanical performances similar to human bone (a low density of 1.74 g/cm^3^ and a low elastic modulus of about 40–44 GPa, very close to that of cortical bone at 30 GPa, [14]) and, during degradation in human environments, it represents an essential nutrient which promotes bone growth and mineralization [8]. The main disadvantage is the risk of more the rapid degradation of magnesium alloy prior to the bone healing process, a fact that requires a difficult co-ordination between the two processes: degradation versus healing [15,16]. Fortunately, the corrosion products that result from the degradation process are non-toxic and can be easily eliminated through the body’s metabolism [15]. However, H_2_ gas, as a corrosion product, can be accumulated in the surrounding tissue as gas bubbles, causing the separation of tissue layers [17,18], and OH^-^ ions, as another possible corrosion product, can produce surface alkalinization and likely the degradation of cells [19]. But in vivo tests showed that the circulation of body fluids facilitates the evacuation of corrosion products that are more abundant in the tissue surrounding the implant in the first period after implantation, and, consequently, the local inflammation is reduced within a matter of days [20,21].

Nowadays, numerous efforts are made to improve the mechanical and biological performances of Mg alloys. One way of action in this direction is the metallurgical optimizing of the chemical composition of the alloy with suitable alloying elements, which allow for obtaining microstructures that are much more adapted to biocompatibility requirements. Another method of action refers to the technological modalities of designing and obtaining a proper bone implant, or even a personalized one.

Referring to the chemical composition, Mg-Zn-Zr alloys (ZK) seem to have superior biocompatibility than already tested Mg-Al-Zn (AZ) or Mg-Zn-RE (WE) [22,23]. Unfortunately, it has been reported that aluminium ions denote a high score for inducing neurotoxicity and brain disorders (Alzheimer’s), and, on the other hand, rare elements, such as Ce, Y and Pr, can induce severe hepatotoxicity even if they can reduce the corrosion rate of these alloys [23]. But for ZK alloys, zinc and zirconium, alongside other possible alloying elements such as Ca, Mn, Si or Ag, arouse real interest in the research world due to their high biosafety [24,25]. Thus, zinc, as one possible alloying element for ZK alloys, has an antimicrobial effect against bacteria in the implantation area [26], being at the same time an important micronutrient for supporting the immune system and enzymatic reactions [27,28]. On the other hand, zinc is reported to improve the mechanical properties of magnesium alloys, decreasing their degradation rate and increasing the osteoblastic cells proliferation during bone reconstruction [28]. When the degradation phenomenon of magnesium alloy occurs, the resulting zinc can be eliminated through the gastrointestinal tract, urine or skin [29,30]. Biological studies have shown that a zinc content of up to 14.5 (%wt.) is beneficial for the human body, above which an unwanted cytotoxicity of the alloy appears [31]. Zirconium, as another important alloying element, is also a biocompatible one, with low ionic toxicity, improving at the same time the corrosion resistance and mechanical properties of the magnesium alloys, even in small amounts [32]. At the same time, zirconium is bioinert if it collects in small amounts in bone or the nervous systems [32]. Calcium, in turn, as a main mineral of bone components [33], represents another potential and important alloying element to magnesium alloys, with a low density (1.55 g/cm^3^) also; research works, such as [34,35], report the exceptional biocompatibility of the binary Mg-Ca alloys. It should also be emphasized that calcium facilitates the hydroxyapatite generation, thereby helping with bone healing [36,37]. Regarding the corrosion resistance of magnesium alloys, calcium, through a controlled composition, can also decrease the corrosion rate, reducing the grain size in the obtained structure [38,39].

Therefore, it would be believed that a promising alternative to the experimental tests carried out so far on ZK ternary alloys (Mg-Zn-Zr) and binary Mg-Ca alloys would be those that include all three alloying elements: Zn, Zr and Ca. Thus, the variant selected for study in the present work has alloying elements with compositions that largely converge with those studied so far, being a new one anyway, and only being reported by the present group of authors. All three alloying elements have a limited solubility in magnesium: zinc, until 6.2%wt. at 340 °C [40]; zirconium, until 0.87%wt. at 700 °C [41]; and calcium, until 1.11%wt. at 521 °C [33]. However, in conformity with [7], if the procedure of the alloy obtaining can induce an extent of these solubilities and reach a non-equilibrium supra-saturated solid solution, without secondary phases, the corrosion resistance of the alloy can be evidently improved. Therefore, for the present study, a similar/close situation/case was considered when selecting the not-studied-before chemical composition Mg-10Zn-0.8Ca-0.5Zr (%wt.). As a consequence, apart from chemical composition designing, new technological modalities of obtaining and processing these alloys in a final fabricated bone implant are planned nowadays, even in a personalised design [42,43]. Standard designs of implants are destinated for large groups of patients. However, to help with the surgeon’s work of ensuring a precise fit for each particular case of patients’ bones, in recent years, what is developing more and more is the trend of the personalization of implants using modern additive manufacturing (AM) procedures. For the next five years, the production of personalized (AM) implants is estimated to reach about USD 70 billion [29].

The alloy selected for study (Mg-10Zn-0.8Ca-0.5Zr (%wt.)) has been proposed and used in a modern AM method, named PBF-LB/M (powder bed fusion-with laser beam/of metal), a method that can assure improved biocompatible and biodegradable implants with custom shaped designs, and with controlled porosities necessary to easily initiate bone regeneration. 

The PBF-LB/M method of additive manufacturing represents a powder bed fusion process which uses a high-density laser beam (L-PBF) on a micro scale [44,45,46]. This method can assure a rapid manufacture of the implants’ metallic parts with custom geometries, without the need for post-processing [31,47]. Therefore, maximum attention should be afforded to the pre-processing step of PBF-LB/M, i.e., the CAD (computer-aided design) of the printed model. The final quality of the obtained product is dictated by the selected process parameters, such as the laser power, scanning speed, distance between the printed layers and distance from the hatch. The PBF-LB/M method can be considered one of the most efficient variants of AM due to its high dimensional accuracy between as-built geometry and the designed geometry, even if some inadvertences can occur like possible balling effects or some misoriented growth in microstructure. In the case of magnesium and its alloys, which are reactive metallic materials, an inert atmosphere of argon must be used. To apply the PBF-LB/M method, the selected alloy must be in powder form, which for this case must be very fine, dense, uniform in size, chemically homogeneous, and as round as possible [48,49,50]. There are several metallurgical methods for obtaining metal powders. One of the more accessible and inexpensive methods is the mechanical alloying process (MA), which involves the milling of pure chemical element powders (≥99.00% purity). Even if there are research works that use the gas-atomization method to obtain powder for additive manufacturing processes, the present work wanted to demonstrate the fact that by using mechanical alloying, it is also possible to obtain an appropriate quality of samples processed through PBF-LB/ m. Using the mechanical alloying method, the obtained powder can attempt a nanocrystalline or even amorphous structure because the milling process implies a multitude of severe plastic deformations and provokes the repeated fracturing and cold welding of the component particles [51,52,53].

For the present study, we proposed a biodegradable alloy, Mg-10Zn-0.8Ca-0.5Zr (%wt.), not tested before by other group of researchers, obtained in a powder state by mechanical alloying, and processed through PBF-LB/M. The aim of the work is to obtain adequate microstructural, mechanical and corrosion characteristics suitable for bone implants. The present study represents a complementary research work to anterior published [54,55] papers, which, at this time, are mainly based on mechanical and corrosion analysis, with a summary microstructural analysis. In addition to the objective stated above, possible research limitations could appear for this incipient experimental program due to the new selected processing route with specific conditions—mechanical alloying followed by PBF-LB/M—applied to a new compositional type of a biodegradable Mg-based alloy.

## 2. Materials and Methods

### 2.1. Mechanical Alloying—First Applied Procedure for Obtaining the Alloy Powder

To obtain the magnesium alloy in powder form, the mechanical alloying method was applied. The chemical elements selected for alloying the magnesium alloy were zinc, zirconium and calcium. In order to carry out the mechanical alloying procedure, chemical elements were used in powder form, with a purity of 99.00%, and an average diameter of the powders as follows: Mg < 100 μm, Zn < 40–50 μm, Zr < 40–50 μm and granules of Ca. The chemical composition calculated to be obtained was Mg-10Zn-0.8Ca-0.5Zr (%wt.). The amounts of alloying elements were chosen in such a way as to agree with those already reported in the specialized literature and discussed above in the Introduction.

**Figure 1:**
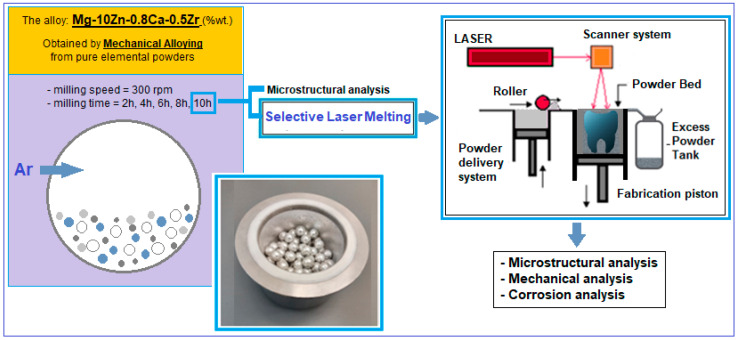
Schema of the experimental program performed for the alloy Mg-10Zn-0.8Ca-0.5Zr.

The applied mechanical alloying procedure (schema in Figure 1—right) involves milling the above powder mixture in the established proportions, using a planetary mill (a PM 100 Retsch type, Haan, Germany) of high-energy, with a capacity of 500 mL and an applied frequency of 50–60 Hz.

The milling speed is usually applied between 150 and 350 rpm; for the present case, a value of 300 rpm was applied. To enhance the milling effect, zirconium oxide balls with a diameter of approx. 10 mm, with a weight ratio of 10:1, were added to the powder mixture. For oxidation protection, the argon atmosphere of 1.5 bar overpressure was used. Also, during the milling process, the cold welding of the powder particles can occur; to prevent this phenomenon, 5% n-heptane solution was added. For the present experimental test, the variable parameter was the milling time. Increasing times from two-to-two hours were tested, starting from 2 h to 10 h. The objective was to finally obtain a powder alloy with a chemical composition and a microstructure as homogeneous and consistent as possible. Therefore, the milling time was extended as much as possible.

After the mechanical alloying procedure, the obtained powder alloy was subjected to a sieving operation with smaller and smaller dimensions; the final one was <30 µm. 

### 2.2. Selective Laser Melting (SLM)—Second Applied Procedure for Obtaining a Bulk Specimen from the Alloy Powder 

The finest powder obtained after mechanical alloying was subjected to AM through the PBF-LB/M method (schema in Figure 1—left). 

For that, the laser used was of the MYSINT 100-3D Selective Laser Fusion type (SISMA s.p.a., Vicenza, Italy), which is a laser special dedicated to printing metallic powder. The applied parameters were the following; laser power—40–150 W; laser speed—300–1000 mm/s; layer height—20–30 µm; volumetric energy density—100–550 J/mm^3^; and inert gas, nitrogen and argon were used. Several samples with dimensions of 10 × 10 × 12 mm (length × width × height) were obtained in order to be further tested in SEM analysis (one sample), compression tests (nine samples) and corrosion tests (three samples).

### 2.3. Microstructural and Mechanical Analysis of the Studied Mg-Alloy Processed by PBF-LB/M

The microstructural analysis of the Mg-Zn-Ca-Zr alloy (in its powder state and after PBF-LB/M processing) started with a SEM-SE (scanning electron microscopy–secondary electron)-imaging investigation that was performed using a Tescan VEGA II-XMU SEM microscope (Tescan Orsay Holding a.s. Brno, Czech Republic). Concomitant calculations of powder characteristics, such as dimension, morphology and homogeneity, were made. The XRD (X-ray diffraction) analysis was performed at room temperature (RIGAKU MiniFlex600, Tokyo, Japan) using Cu-Kα radiation with a scattering angle 2θ in the range of 30–90 degrees for a step size of 0.02 degrees. Nine samples of the studied Mg-Zn-Ca-Zr alloy, after the PBF-LB/M printing procedure, were subjected to the compression test by using a universal testing machine of the INSTRON 3382 type (Instron Ltd., High Wycombe, Buckinghamshire, HP123SY, Birmingham, UK). The samples were subjected to constantly increasing loads until they finally broke.

### 2.4. Corrosion Analysis of the Studied Mg-Alloy Processed by SLM

The corrosion monitoring was carried out using a potentiostat (Radiometer Analytical VoltaLab PGZ 402, Lyon, France). Three corrosion tests were conducted: open-circuit potential, electrochemical impedance spectroscopy (EIS), and polarisation for corrosion (Tafel plots) [56]. The open-circuit-potential tests were determined for the studied Mg alloy; EIS was measured using a value that was 200 mV more electropositive than that determined during open-circuit-potential tests (AC signal impedance acquisition was performed for EIS tests, using an AC sine wave amplitude of 2 mV), and the polarisation for the corrosion test was performed using a range from −1600 mV to +1600 mV and a scan rate of 2 mV/s; the potentiostat’s software (VoltaMaster 4, version 7.9) provided the following data, using a preset calculation method (1st Stern method: Tafel): the corrosion rate (V_corr_), the corrosion current (i_corr_), the polarization resistance (R_p_), the electrode potential (E_(i=o)_) and the anodic and cathodic Tafel constants (β_a_ and β_c_). The tests were performed in triplicate.

## 3. Results and Discussions

### 3.1. Microstructural Characterization of the Mg-Zn-Ca-Zr Alloy in Powder State and after PBF-LB/M Processing

As already stated in the Introduction, in the previously published articles [54,55] the preponderance was the microstructural analysis of the studied alloy. In the article [54], the emphasis was placed on the step-by-step follow-up of the morphological and compositional evolution of the powder alloy obtained through mechanical alloying by varying the milling time. In the second article [55], the microstructural evolution of the subsequent AM samples using the PBF-LB/M method by varying the volumetric energy-density parameter was highlighted. The present work tries to take a step forward by deepening the research regarding the mechanical behaviour of the same alloy processed by PBF-LB/M as well as the corrosion resistance of this alloy. Thus, the microstructural analysis was performed, but this time, only on the samples that showed the best previous results: first, on the alloy powder obtained through mechanical alloying, with a milling time of 10 h; then, on the samples obtained after PBF-LB/M processing, applying a volumetric energy density (VED) of 134 J/mm^3^ and a scanning speed of 600 mm/s, as two parameters with important influence.

**Figure 2:**
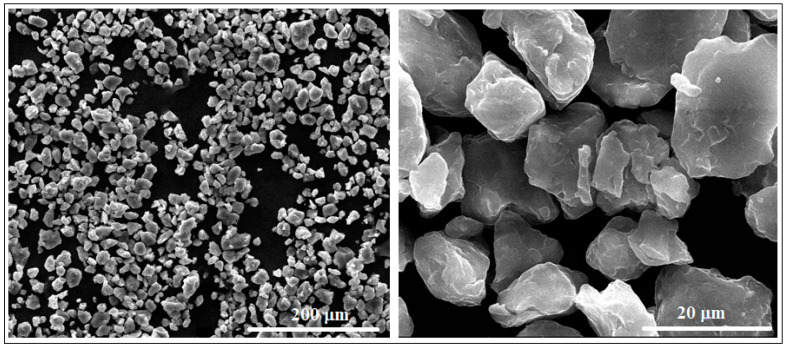
The SEM-SE images for the Mg-Zn-Ca-Zr powder alloy after mechanical alloying with a milling time of 10 h.

Figure 2 shows two representative SEM images obtained after microscopic analysis of the studied Mg-Zn-Ca-Zr alloy in its powder state, as it has resulted from the mechanical alloying procedure with a milling time of 10 h. Looking at the powder morphology, several important features were observed using a SEM-calibrated microscope.

First, it is a dimensional homogeneity of the powder particles with an average value of 16.2 ± 8.6 µm. In conformity with [42,43,44], this average dimension is satisfactory for the subsequent PBF-LB/M procedure.

**Figure 3:**
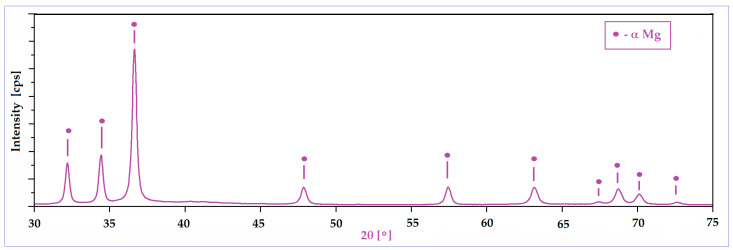
The XRD spectra of the Mg-Zn-Ca-Zr powder alloy after mechanical alloying with a milling time of 10 h.

A second important characteristic refers to the surface of the powder particles that looks to be homogeneous, with a uniform colour and without signs of inhomogeneities; the structure consists of a single phase, with alloying elements completely solubilized in magnesium and, consequently, without secondary phases. Moreover, the lack of secondary phases and the complete solubilization of the alloying elements was also proven by XRD analysis (Figure 3), which indicates patterns corresponding to the magnesium-based solid solution, α-Mg. Keeping in mind that for the present case, the amount of zinc exceeds the limit of solubility in Mg (6.2%wt.), if the synthesis procedure had followed the classic line of alloy obtaining (melting–solidification) then the secondary phase MgZn_2_ should have been formed. Unlike the classic version, during the mechanical alloying procedure, there are severe plastic deformations of the powder particles, accompanied by cold welding and their strong and constant fractures, which give rise to a strong energy that can lead to a homogeneous distribution of the chemical elements in the Mg-powder particles. There are works, such as [57], which clearly confirm this possibility, and even for the extreme case where the amorphous structure can be obtained [58] at much longer milling times, about 70 h or more. In the present case, the total absence of MgZn_2_ in the XRD patterns cannot be absolutely guaranteed for the real structure, considering the possible minute amount of precipitate formed, and the low sensitivity of the device used.

Finally, another positive morphological characteristic observed was that, unlike the shorter milling times for which sharper shapes of the alloy powders are observed, at 10 h, no agglutinated particles were observed, having an individualized appearance, with regular, smooth and relatively rounded shapes. Therefore, all these particular characteristics can be considered to have been suitable for the subsequent PBF-LB/M process for which, in general, a fine powder with a homogeneous and almost rounded shapes is required.

**Figure 4:**
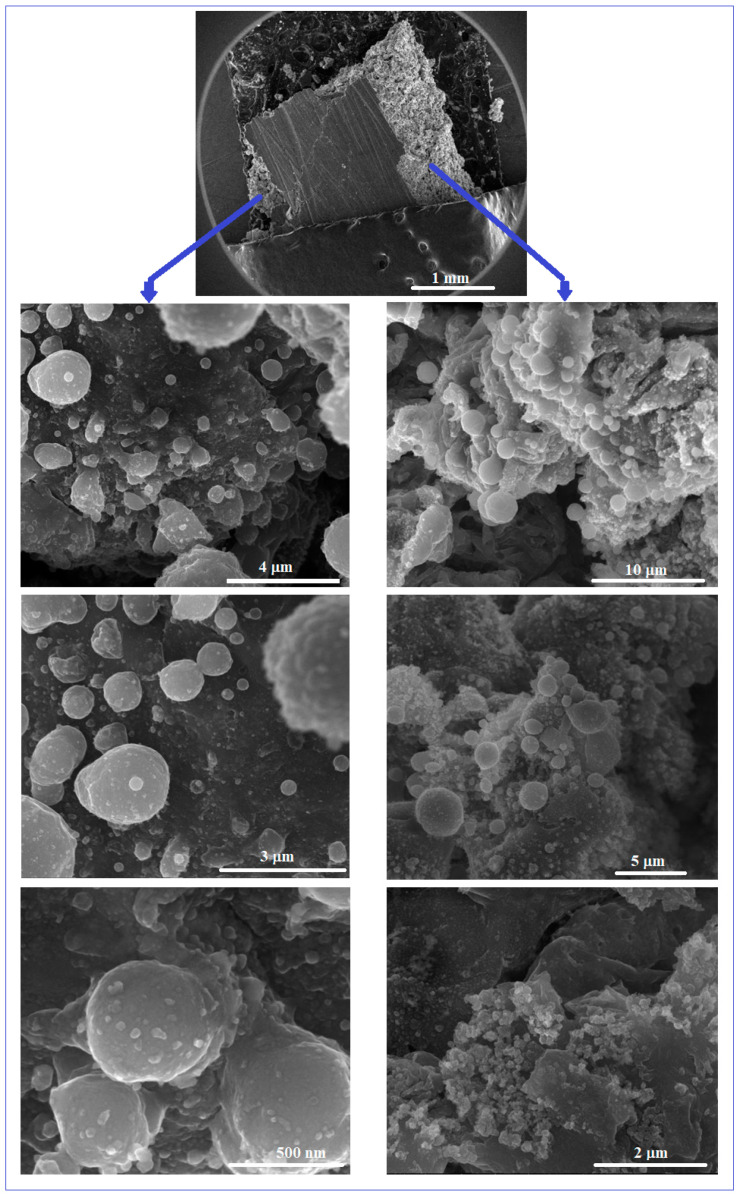
The SEM-SE images for the Mg-Zn-Ca-Zr alloy after PBF-LB/M processing.

Figure 4 shows some representative SEM images obtained by the microscopic analysis of the Mg-Zn-Ca-Zr alloy after the PBF-LB/M procedure. The initial macroscopic examination of the obtained PBF-LB/M samples of 10 × 10 × 12 mm shows a robust state, but is relatively porous and without cracks that correspond to the proposed objective—to obtain a structure that can reproduce that of cortical bone.

In that sense, the examination by SEM analysis was made inside of the PBF-LB/M samples, in order to observe the homogeneous distribution of the robustness and porosity, cumulatively, in the entire volume of the samples. From the SEM images corresponding to Figure 4, it can be seen that the examined surfaces, exposed to a gradually increasing magnification, show a dense and unbreakable morphology, without the balling effect, known to be detrimental to the mechanical performance and therefore undesirable.

The degree of porosity obtained by the PBF-LB/M procedure must be correlated with a real and therefore desired level of cortical bone porosity. This “desired” level can be set/standardized depending on the type and location of the bone implant and can be achieved by a judicious selection of PBF-LB/M processing parameters. However, for the present case, the PBF-LB/M samples, judging by their morphological condition, can be considered suitable for an early examination of their mechanical and corrosion performance.

### 3.2. Mechanical Characterization of the Mg-Zn-Ca-Zr Alloy in Its Powder State and after PBF-LB/M Processing

**Figure 5:**
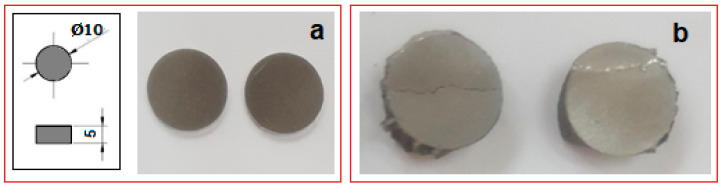
Macroscopic views of the PBF-LB/M samples (**a**) before and (**b**) after compression tests.

**Figure 6:**
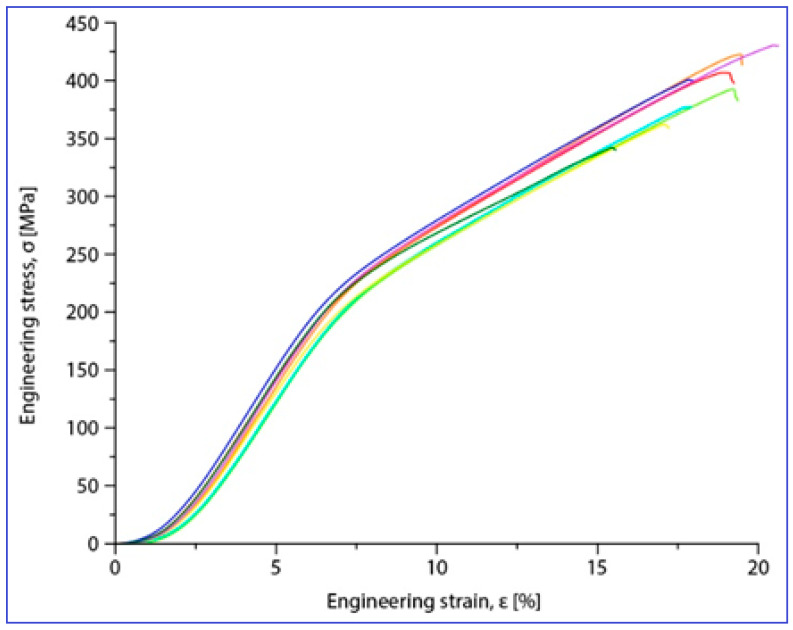
Stress–strain diagrams for the nine PBF-LB/M samples tested in compression tests.

**Figure 7:**
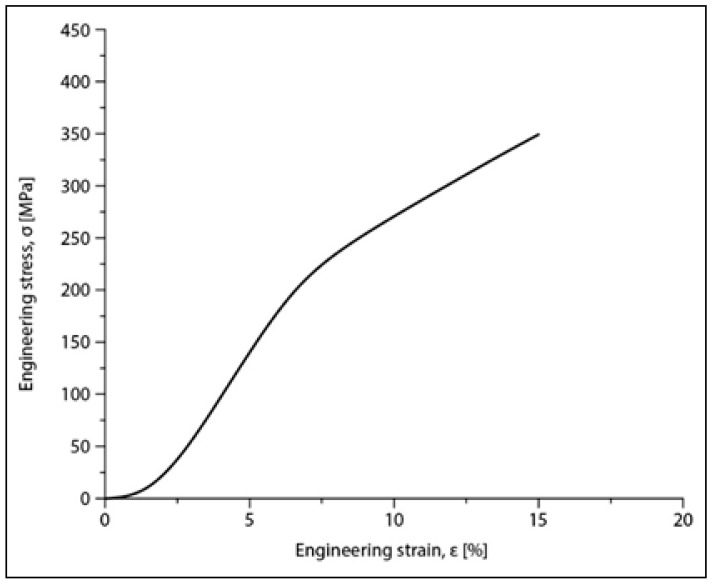
Stress–strain diagram for the Mg-Zn-Ca-Zr alloy in the PBF-LB/M condition after compression tests.

Compression tests were applied for a set of nine samples realized by PBF-LB/M processing. Figure 5 shows the macroscopic dimensional aspect of some samples, before and after testing. The strain–stress curves for each of all nine tested samples are presented all together in Figure 6. After processing the data resulting from these curves, the determination of the mechanical characteristics was made for all tested samples. Thus, Figure 7 shows the representative strain–stress curve resulting from weighting the data from all tested samples, and Table 1 shows the average corresponding values for the maximum compressive strength (σ_max_), deformation to fracture (ε_max_) and elastic modulus (E). The standard deviation was calculated and has been included in Table 1.

Examining the data in Table 1, it can be said that the values obtained for the main mechanical properties are generally close to those of cortical bone [8,9], especially for the Young’s modulus (30 GPa for cortical bone compared to 42 GPa for the tested samples). This means that, for an implant made of this alloy, using PBF-LB/M technology that ensures a certain desired porosity of the material, the risk of the stress-shielding phenomenon is very low, especially if the implant is designed to be biodegradable over time.

### 3.3. Corrosion Analysis of the PBF-LB/M-Processed Mg-Based Alloy

**Figure 8:**
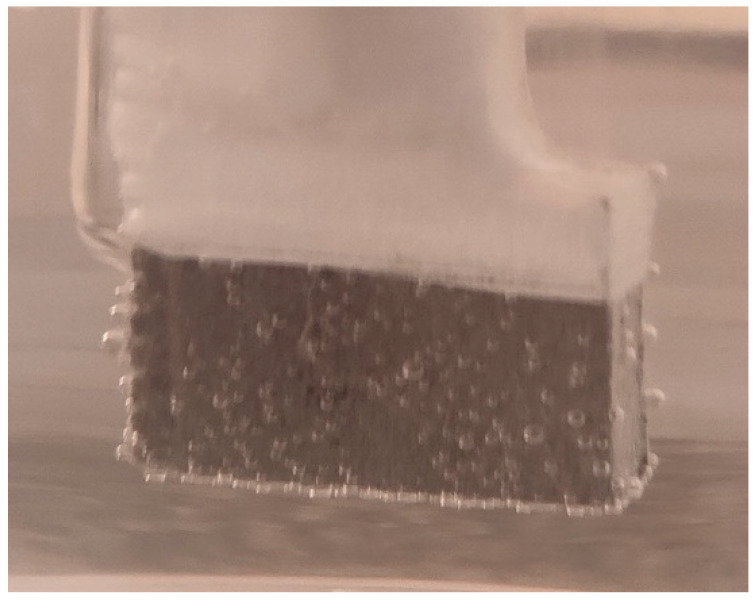
Hydrogen bubbles released on the surface of the working electrode (sample).

For the corrosion test, three samples of studied Mg alloy after AM using the PBF-LB/M procedure were used. These three samples were tested in PBS (Phosphate-buffered saline) solution with three different pH values (based on Roth Roti^®^CELL PBS CELLPURE^®^, having the composition 154.004 mM NaCl, 5.599 mM Na_2_HPO_4_, 1.058 mM KH_2_PO_4_), with the intention of covering the widest possible range of pH variation in the human body: (1) stock PBS [59] solution with a pH of 7.4; (2) HCl (0.1M)-doped PBS with a pH of 3.16 and (3) KOH (pellets)-doped PBS with a pH of 10.1. All tests were conducted using Ag/AgCl (3M KCl) as a reference electrode, a Pt wire electrode as counter electrode, and the samples as the working electrode. For each pH, three different immersion times were tested: 0 h, 24 h and 48 h. During all performed tests, an important amount of hydrogen emission was observed—Figure 8. After the corrosion tests were concluded, each of three electrochemical cells were stored in a digital drying oven at 37 °C.

**Figure 9:**
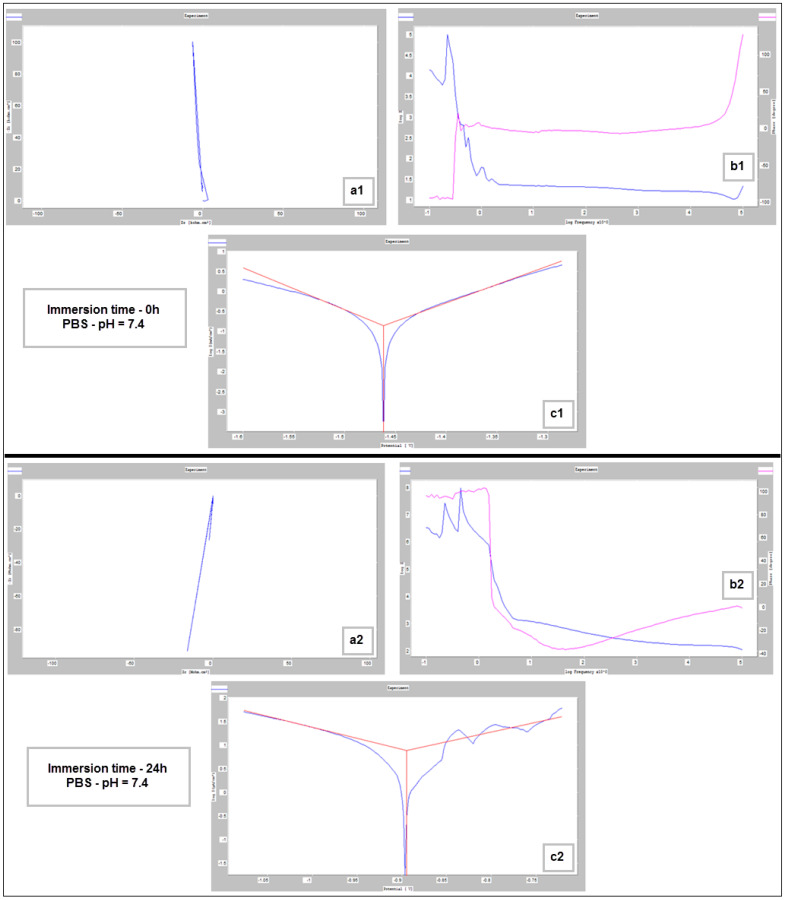
Nyquist (**a1**,**a2**), Bode (**b1**,**b2**) and Tafel (**c1**,**c2**) diagrams for the PBF-LB/M sample tested in PBS with pH 7.4, at immersion times of 0 h and 24 h.

**Figure 10:**
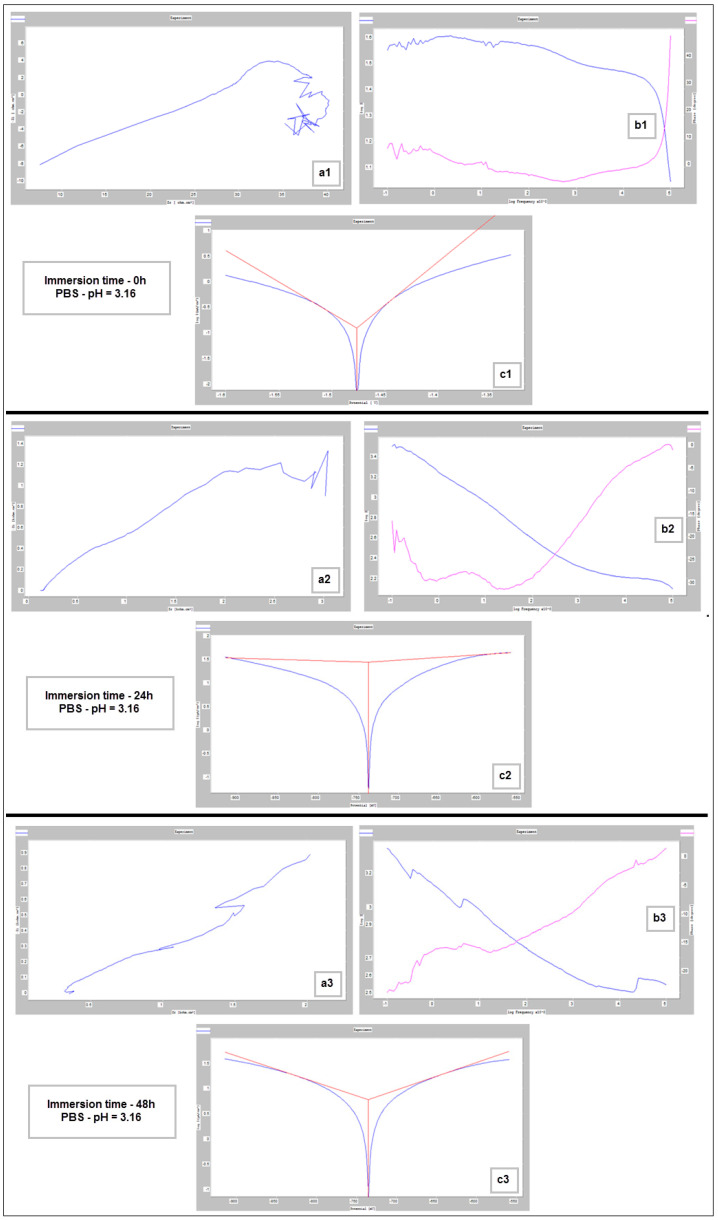
Nyquist (**a1**–**a3**), Bode (**b1**–**b3**) and Tafel (**c1**–**c3**) diagrams for PBF-LB/M sample tested in PBS with pH 3.16, at immersion times of 0 h, 24 h and 48 h.

**Figure 11:**
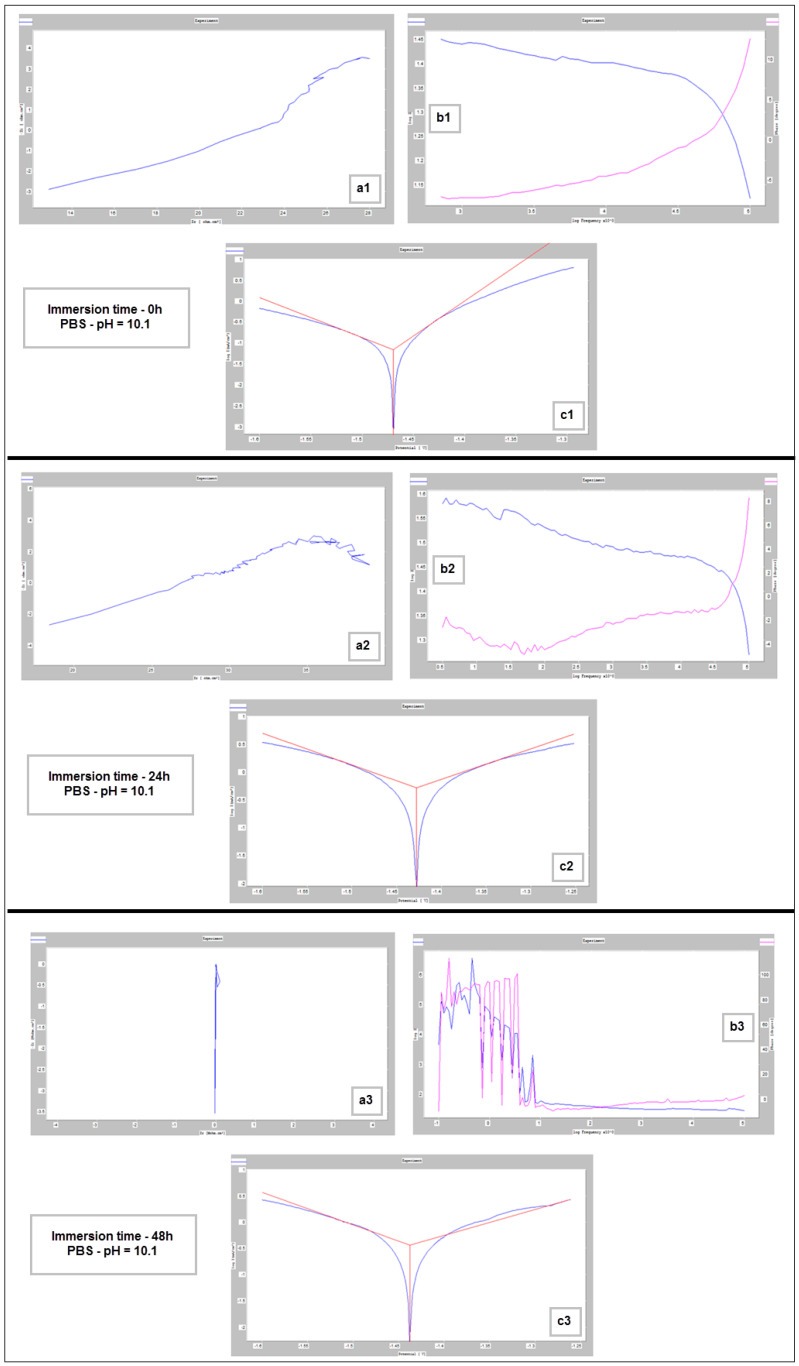
Nyquist (**a1**–**a3**), Bode (**b1**–**b3**) and Tafel (**c1**–**c3**) diagrams for PBF-LB/M sample tested in PBS with pH 10.1, at immersion times of 0 h, 24 h and 48 h.

Figure 9, Figure 10 and Figure 11 show the Nyquist, Bode and Tafel spectra obtained for the tested samples. The resulting Nyquist spectra have a convulsing/trembling shape due to the noise generated by the massive amounts of hydrogen release. These hydrogen bubbles adhered to the surface of the sample and, as such, interfered with the readings during the tests performed, as seen in Figure 8. The generation of hydrogen, inevitable in the case of magnesium alloys, can create a considerable variability in the results, unfortunately; this situation can be augmented, in addition, by the high level of roughness of the surface of the PBF-LB/M samples, a sine qua non condition, by which the generation of hydrogen is higher than in the case of the more compact surfaces of the samples. This roughness is assumed in this case, out of the desire to imitate the more porous surface of the cortical bone that will support the joining with a biodegradable implant made of such a magnesium alloy. Being an accelerated corrosion test, finally, all samples were dissolved and formed a dark grey precipitate on the bottom of their respective electrochemical cells.

**Figure 12:**
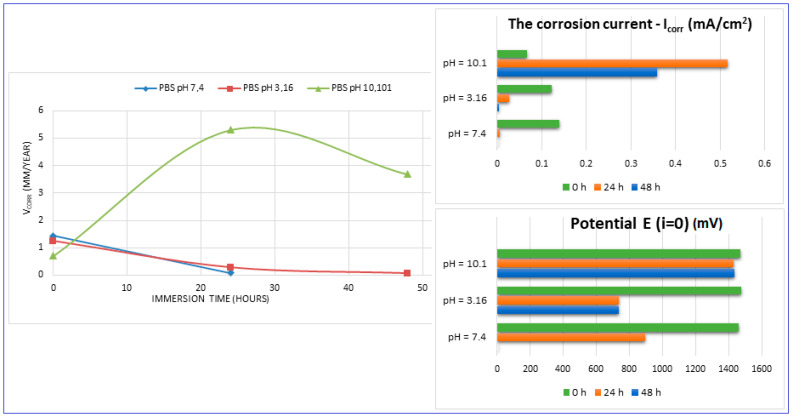
The evolution of the corrosion rate for the tested samples.

The main parameters determined, which resulted from these corrosion analyses, are shown in Table 2: the corrosion current (j_corr_), the electrode potential (E_(i=o)_) and the corrosion rate (V_corr_). The resulting corrosion-rate values for each tested sample are indicated in Figure 12.

For increasing the corrosion performance of a metallic alloy, it was generally established that the electrode potential must have an upward trend (it should be high), and the corrosion current must be low [60,61,62]. This fact can be seen from the values of Table 2. A similarity in the corrosion parameters’ evolution can be seen for both neutral and acidic pH. If we compare these values with those corresponding to the commercial ZK60 alloy (around 1.45 mm/year [63,64,65,66,67,68]), it can be found that the values obtained here can be considered quite satisfactory.

For basic pH (10.1), however, notable differences are observed: the electrode potential and the current density have a fluctuating variation (increase/decrease), and the resulting corrosion rates are outside the ranges generally reported. This fact is highlighted by Figure 12 also. These types of inadvertences can probably be attributed to the fact that the hydrogen release was not measured. It should be pointed out that the PBS solution with a basic pH (10.1) represents an experimental situation and not a real physiologic test, as done for real regular implants. However, the alkalization can occur in the case of Mg-based implants reaching a pH level of ten and even higher [69,70]. Moreover, in conformity to [7], it seems that the corrosion rate can be substantially improved by expanding the solubility of the alloying elements using non-equilibrium techniques, without causing the serious micro-galvanic corrosion that usually is generated when the added alloying elements’ precipitates as second-phase particles. The mechanical alloying procedure, used for the present experimental program, can be considered a non-equilibrium technique, because the amount of zinc exceeds the limit of solubility in Mg (10%wt. instead of 6.2%wt.) through severe plastic deformations that occur during the process.

However, as a good result noted from these experiments, it should be mentioned and emphasized that at a neutral pH, the corrosion rate of the Mg-10Zn-0.8Ca-0.5Zr alloy (1.426 mm/year) is very close to that of the alloy considered an etalon for comparison with nowadays —ZK 60 (1.45 mm/year) [63,64,65,66,67,68].

## 4. Conclusions

A magnesium alloy with the composition of Mg-10Zn-0.8Ca-0.5Zr (%wt.) has been obtained via mechanical alloying using pure elemental powders. After 10 h of milling in an argon atmosphere, the obtained powder alloy reveals a uniform dimensional homogeneity with an average particle size of 16.2 ± 8.6 µm and no agglutinated particles. The powder particles have also a homogeneous microstructure, with a uniform colour and no agglutinated particles. This powder alloy has been processed by additive manufacturing using the PBF-LB/M method.

The SEM microstructural analysis of the PBF-LB/M samples reveal a dense and unbreakable morphology, but with a porosity able to reproduce that of cortical bone, and without the undesirable balling effect.

After compression tests, the alloy samples in the PBF-LB/M condition have a Young’s modulus of 42 GPa, close to that of cortical bone, 30 GPa, avoiding the stress-shielding effect.

The PBF-LB/M samples tested for corrosion resistance in PBS solution with three different pH values show that the corrosion parameters have a satisfactory evolution. At the normal neutral-body pH, the corrosion rate of the Mg-10Zn-0.8Ca-0.5Zr alloy (1.426 mm/year) is very close to that of the ZK 60 alloy (1.45 mm/year).

## Figures and Tables

**Table 1:** Mechanical properties of the studied alloy after PBF-LB/M processing: ultimate compressive strength (σ_max)_; deformation to fracture (ε_max_); elastic modulus (E); SD (Standard Deviation).

The Alloy in PBF-LB/M Condition	σ_max_ [MPa]	ε_max_ [%]	E [GPa]
Mg-10Zn-0.8Ca-0.5Zr (%wt.)	381.25 ± 17.19	17.92 ± 0.44	42.10 ± 1.08

**Table 2:** Main corrosion parameters for tested PBF-LB/M samples in PBS solution, with three different pH values and different immersion times.

Mg-Alloy, PBF-LB/M-Processed, Immersed in PBS with pH:	Immersion Time (h)	V_corr_ (mm/Year)	I_corr_ (mA/cm^2^)	Potential E(i=o) (mV)
pH = 7.4	0	1.426 ± 0.04	0.139 ± 0.026	−1462.1 ± 2.1
	24	0.079 ± 0.01	0.007 ± 0.001	-895 ± 1.3
	48	-	-	-
pH = 3.16	0	1.252 ± 0.04	0.122 ± 0.016	−1476.4 ± 2.1
	24	0.278 ± 0.02	0.027 ± 0.004	−737.9 ± 1.1
	48	0.060 ± 0.01	0.005 ± 0.001	−735 ± 1.1
pH = 10.1	0	0.703 ± 0.06	0.068 ± 0.002	−1469.6 ± 2.8
	24	5.305 ± 0.12	0.517 ± 0.022	−1428 ± 2.2
	48	3.679 ± 0.10	0.359 ± 0.013	−1437.6 ± 2.4

## Data Availability

The data presented in this study are available on request from the corresponding author.
